# Sex-specific effects of therapeutic hypothermia on short- and long-term microglial and astrocytic reactivity after severe neonatal hypoxia–ischemia

**DOI:** 10.1007/s11011-026-01957-y

**Published:** 2026-08-29

**Authors:** Ricardo Ribeiro Nunes, João Vitor Miotto Tauffer, Bianca Büchele Martins, Isadora D’Ávila Tassinari, Carlos Alexandre Netto, Ana Helena Paz, Luciano Stürmer de Fraga

**Affiliations:** 1 Graduate Program in Biological Sciences: Physiology, Instituto de Ciências Básicas da Saúde (ICBS), Universidade Federal Do Rio Grande Do Sul (UFRGS), Porto Alegre, Brazil; 2 Department of Physiology, ICBS, UFRGS, Porto Alegre, Brazil; 3 Department of Biochemistry, ICBS, UFRGS, Porto Alegre, Brazil; 4 Department of Morphological Sciences, ICBS, UFRGS, Porto Alegre, Brazil; 5 Center of Experimental Research, Hospital de Clínicas de Porto Alegre (HCPA), Porto Alegre, Brazil

**Keywords:** Neonatal hypoxia–ischemia, Therapeutic hypothermia, Neuroinflammation, Microglia, Astrocytes, Sexual dimorphism

## Abstract

Therapeutic hypothermia (TH) is the standard treatment to reduce mortality and neurological impairments after neonatal hypoxia–ischemia (HI). However, TH does not fully suppress neuroinflammation, leading to persistent neuroinflammatory responses that may exacerbate brain injury and limit TH efficacy. In addition, the influence of sexual dimorphism on these outcomes remains poorly understood. We investigated short- and long-term microglial and astrocytic reactivity in male and female neonatal rats following HI and TH treatment. At postnatal day 7 (PND7), male and female pups underwent right common carotid occlusion followed by exposure to hypoxia (8% O_2_). TH was induced by reducing body temperature to 32°C for 5 h. Brains were collected at PND8 or PND21 to assess brain injury volume, brain water content, and microglial and astrocytic reactivity based on Iba-1 and GFAP mean fluorescence intensity. Behavioral impairments were evaluated using negative geotaxis, olfactory discrimination, and cylinder tests. TH did not reduce injury volume but attenuated neuronal cell loss in males and reduced brain edema in females. In males, microglial reactivity was reduced by TH at PND8 and PND21 and astrocytic reactivity only at PND8. Olfactory discrimination was impaired in females and prevented by TH, whereas motor performance deficits and developmental impairments were not prevented by TH. Our results reinforce that the neuroprotective efficacy of TH is limited in severe HI injury and that, despite reducing early neuroinflammation, TH fails to prevent long-term neuroinflammatory responses. Notably, the early anti-inflammatory effects were only observed in males, underscoring the need for additional therapeutic strategies, particularly for females.

**Graphical Abstract:**

**Figure:**
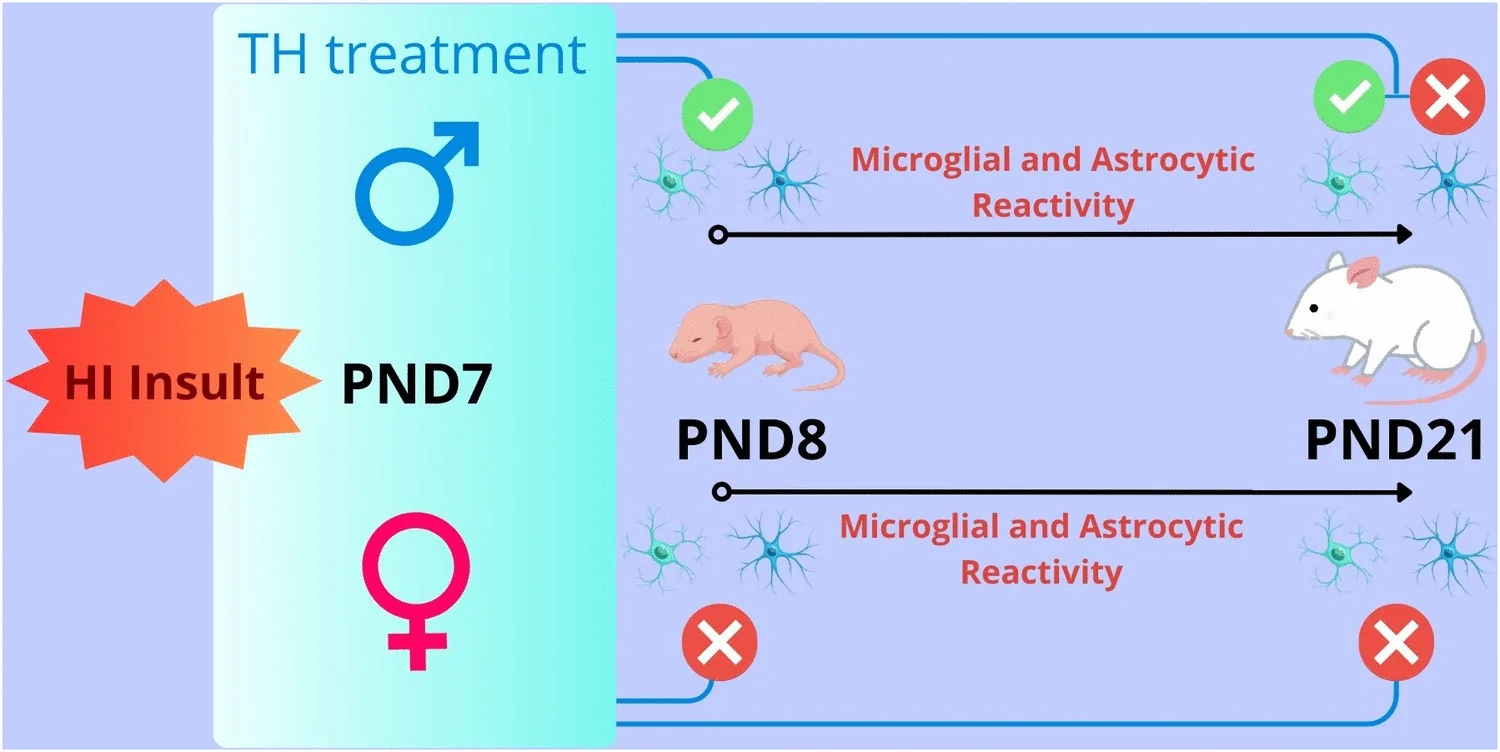

**Supplementary Information:**

The online version contains supplementary material available at https://doi.org/10.1007/s11011-026-01957-y.

## Highlights

TH did not effectively ameliorate severe HI-induced brain injury.

Microglial and astrocytic reactivity was higher in males.

Short- and long-term microglial and short-term astrocytic reactivity were reduced by TH in males.

TH attenuated olfactory discrimination impairment in females.

Motor and developmental impairments were not reduced by TH.

**Supplementary Information:**

The online version contains supplementary material available at https://doi.org/10.1007/s11011-026-01957-y.

## Introduction

Since 2010, therapeutic hypothermia (TH) has been established as the standard treatment for newborns suffering from hypoxic-ischemic encephalopathy (Karnatovskaia et al. 2014), a condition that is responsible for a significant number of neonatal deaths and neurological disabilities worldwide. The reduction of body temperature to approximately 33.5°C for 72 h has been shown to reduce mortality and improve both short and long-term neurodevelopmental impairments (Wassink et al. 2019). Despite these benefits, subsequent clinical and experimental studies revealed important limitations of TH: its efficacy depends on being initiated within a narrow therapeutic window of about six hours after the insult (Fabres et al. 2022; Sabir et al. 2012), it is less effective in cases of severe hypoxia–ischemia (HI) (Wood et al. 2016), and its effectiveness and safety remain uncertain in preterm babies—a population at high risk for HI (El-Dib et al. 2025). We have previously shown limitations of TH in reducing HI-induced behavioral and developmental impairments in rats (Nunes et al. 2024), although brain injury and cell death were reduced (Zang et al. 2023). These TH limitations have led to the search for new adjunctive or alternative therapies, as well as for a deeper understanding of the mechanisms underlying these restrictions in TH efficacy (Zhou et al. 2020).

Among the mechanisms associated with brain injury in neonatal HI, some effects of TH in neuroinflammatory parameters have been investigated. It can reduce pro-inflammatory cytokines in the brain tissue, such as interleukin-1 beta (IL1-β), tumor necrosis factor alpha (TNF-α), and interleukin-6 (IL-6) (Fabres et al. 2025; Yuan et al. 2014). TH can also reduce pro-inflammatory microglial polarization and migration to injury sites (Seo et al. 2012). It has also been associated with the reduction of the complement pathway (Shah et al. 2021) and the reduction in astrogliosis (Fabres et al. 2025, 2022). Despite these beneficial effects, TH is unable to completely suppress neuroinflammation, which can persist after rewarming (Zhou and Davidson 2023). Since TH must only be applied immediately after the hypoxic insult, these persistent neuroinflammatory processes cannot be directly targeted. Chronic neuroinflammation is responsible for brain injury progression days to months after the HI event (Ziemka-Nalecz et al. 2017), establishing an important therapeutic target.

Sex-specific responses to HI should also be considered when investigating outcomes and treatment efficacy (Kelly et al. 2023). Males are known to present increased brain injury compared to females (Al Mamun et al. 2018; Mirza et al. 2015). In our previous meta-analysis, we found that males show greater astrocyte and microglial reactivity and leukocyte infiltration (Nunes et al. 2025). Females are more protected from neuroinflammatory damage since they display greater anti-inflammatory responses after HI, such as reduced microglial reactivity (Villapol et al. 2019) and increased Treg response (Beckmann et al. 2022). Understanding whether neuroinflammation after HI differs between sexes is therefore crucial, not only for clarifying mechanisms but also for optimizing therapeutic strategies such as TH.

Evidence indicates that the effects of TH on neuroinflammation are heterogeneous and only partially beneficial, highlighting their dependence on time and severity. The role of TH oin long-term neuroinflammation still needs to be fully elucidated to better understand the limitations of TH and to guide the development of complementary immunomodulatory therapies targeting chronic neuroinflammation. Additionally, the effects of sex differences in this context have not been investigated. Therefore, this study aimed to evaluate the effects of therapeutic hypothermia after severe neonatal HI on short- and long-term microglial and astrocytic reactivity in the injured brain and to determine whether these responses are sex-specific.

## Methodology

### Animals and ethics

A total of 261 Wistar rat pups of both sexes, at postnatal day 7 (PND7), were used in this study. Animals were obtained from the Animal Breeding Center (CREAL, *Centro de Reprodução e Experimentação de Animais de Laboratório*) of the Federal University of Rio Grande do Sul (UFRGS) and transported at PND2 to the Animal Facility (UEA, *Unidade de Experimentação Animal*) of the Hospital de Clínicas de Porto Alegre (HCPA), where they were allowed to acclimate for five days. Each litter consisted of nine pups (four females and five males), from which one male was designated as a sentinel animal, as described in the “Therapeutic Hypothermia” section. Pups were kept with their respective dams throughout the study and maintained in plexiglass cages (49 × 34 × 16 cm) with the floor covered by standard bedding. Cardboard paper rolls and sheets of paper were provided for nesting and sheltering. Animals were kept under controlled conditions with a 12 h light/dark cycle and room temperature of about 22°C. Dams received food and water ad libitum.

This study was approved by the Institutional Animal Care and Use Committee of HCPA (#2022–0598). All procedures were carried out in accordance with the Animal Research: Reporting of In Vivo Experiments (ARRIVE) 2.0 guidelines and the U.S. Public Health Service Policy on Humane Care and Use of Laboratory Animals, revised in 2015.

### Hypoxia–ischemia

**Fig. 1:**
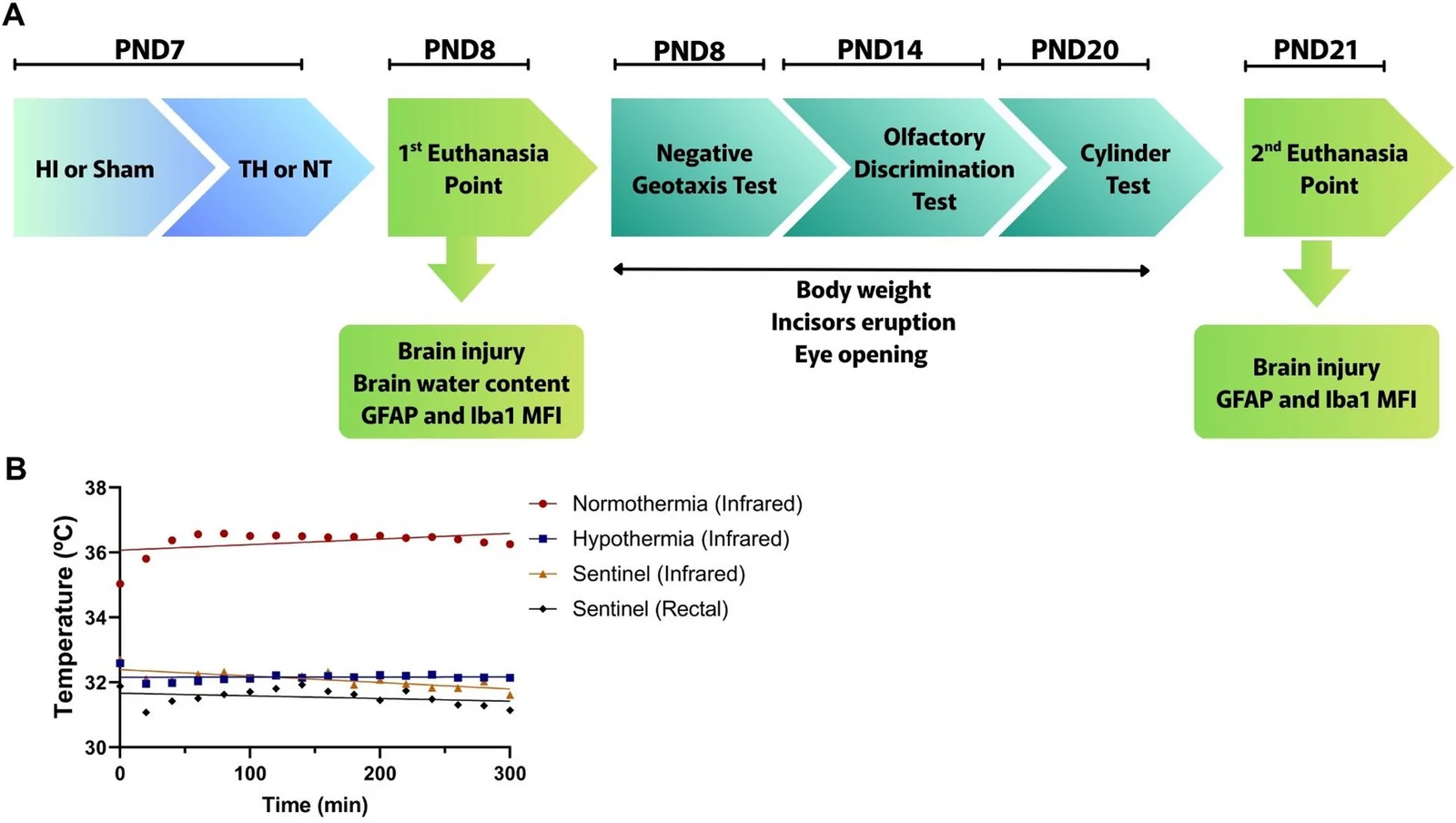
(**A**) Experimental design. At PND7, pups were submitted to the HI model, which consists of the occlusion of the right common carotid artery followed by exposure to a hypoxic atmosphere with 8% oxygen for 90 min. The SHAM groups were submitted to sham surgery and remained in normoxic conditions. Animals were treated with TH by reducing their body temperatures to 32 °C for 5 h, while non-treated animals were maintained in normothermic conditions. At PND8, one group of animals was euthanized for the respective evaluations. Another group of animals was accompanied until PND21 and evaluated by the indicated behavioral tests at different ages, and developmental parameters were verified daily. At PND21, this group was euthanized for the respective evaluations. (**B**) Body temperature of PND7 rats submitted to 5 h hypothermia (SHAM + TH and HI + TH, body temperature around 32 °C) or normothermia (SHAM and HI, body temperature around 36 °C), including both male and female rats. Temperature was measured with an infrared thermometer every 20 min. Sentinel male pups were submitted to hypothermia and had their rectal temperature measured for treatment temperature control but also had their body temperature measured by an infrared thermometer for instrument comparison. Results are presented as mean ± SEM. HI: hypoxia–ischemia; MFI: mean fluorescence intensity; NT: normothermia; PND: post-natal day; TH: therapeutic hypothermia

The Rice-Vannucci model (Rice et al. 1981) was performed in male and female pups at PND7 (Fig. 1A) based on our previous studies (Nunes et al. 2024; Spies et al. 2025; Tassinari et al. 2024; Zang et al. 2023). Briefly, the pups were anesthetized with isoflurane (5% for induction and 3% for maintenance), and the right common carotid artery (CCA) was permanently occluded. Animals from the SHAM groups underwent sham surgery, in which the CCA was exposed but not occluded. Group assignment was performed randomly using a dice-based method by throwing a four-sided die (one number for each experimental group: SHAM, SHAM + TH, HI and HI + TH) and an eight-sided die (one number for each pup of the litter). If the die returned a number that had already been assigned, the next available number was considered. Following surgery, animals were allowed to recover for one hour before being exposed to a hypoxic atmosphere (8% oxygen) for 90 min in a hypoxic chamber. The chamber was placed inside a neonatal incubator (Fanem® C186TS) maintained at 33 °C, in order to keep the pups’ body temperature close to 37°C. Animals from the SHAM groups were kept in another incubator at 33 °C for the same period. Pups were then returned to their dams for an additional 90-min recovery period.

### Therapeutic hypothermia

For hypothermia induction, the pups were placed individually in glass beakers fixed to the bottom of a water bath (Precision Scientific® Dubnoff), which allowed their body temperature to be reduced to 32 °C for 5 h (Wood et al. 2016). The rectal temperature of a sentinel pup, which had not undergone HI or SHAM surgery, was continuously checked to monitor body temperature, and the water bath temperature was adjusted accordingly (Gundersen et al. 2023). Rectal temperature was measured with a microprobe thermometer (Physitemp® BAT-12), which was inserted into the rectum and attached along the sentinel animal’s tail with tape, allowing the pup to move freely without risk. Sentinel animals were euthanized by anesthetic overdose immediately after the hypothermia protocol and were not included in any analysis. Additionally, an infrared thermometer (Incotherm® TCI-1000) was used to monitor the scalp temperature of all animals every 20 min, including sentinels. Animals that were not exposed to hypothermia remained for the same 5-h period inside a neonatal incubator (Fanem® C186TS) at 33 °C, in order to maintain their body temperature at 37 °C. At the end of hypothermia, the pups were placed together with their littermates inside the neonatal incubator, resulting in a rewarming rate of approximately 1 °C every 5 min. After 20 min of rewarming to approximately 36 °C, pups were returned to their dams.

### Histological processing

Pups were euthanized at either PND8 or PND21 for evaluation by Nissl staining and immunofluorescence. Euthanasia was performed under isoflurane anesthesia, followed by thoracotomy and catheter placement in the left ventricle/aorta. Transcardial perfusion was carried out with 0.9% saline (1 mL/g) and 4% paraformaldehyde (2 mL/g). The brain was dissected, post-fixed overnight in 4% paraformaldehyde, and then stored in 70% ethanol. Tissues were dehydrated through increasing ethanol concentration series, cleared in xylene, and embedded in paraffin. Serial sections were cut at a thickness of 7 µm using a microtome (Microm® HM325) and collected on gelatin-coated slides. Sections were collected from the posterior hippocampus to the posterior olfactory bulb, with an interval of 105 µm for PND8 (corresponding to 1 out of every 15 sections) and 154 µm for PND21 (corresponding to 1 out of every 22 sections).

### Brain injury assessment

For each animal, a total of seven slides containing five sections each (representing the brain from the posterior olfactory bulb until the posterior hippocampus) were used in brain injury volume analysis and cell counting. Slides containing the brain sections were deparaffinized in xylene, rehydrated through decreasing ethanol concentration series and stained with 0.1% cresyl violet with 0.3% glacial acetic acid at 40 °C. Stained sections were then differentiated in 70% and 95% ethanol, dehydrated in 99% ethanol, cleared in xylene, and coverslipped with Canada balsam.

Slides were scanned for whole-hemisphere analysis of brain injury volume. The total areas of the left and right hemispheres, as well as the non-injured area of the right hemisphere (ipsilateral to carotid occlusion) were measured using ImageJ software (Schneider et al. 2012). Measurements were performed in grayscale images with enhanced contrast, for better differentiation between the injured and the healthy regions. The areas were converted to volumes by multiplying the area by the respective section interval length (105 µm or 154 µm). Infarct volume percentage was calculated as: ((total left hemisphere volume—healthy right hemisphere volume)/total left hemisphere volume) × 100 (Sun et al. 2015). At PND8, the degree of atrophy was also analyzed by using the same formula described above while replacing the healthy volume with the total volume of the right hemisphere. This analysis was not done at PND21 because all injured tissues corresponded to atrophic areas.

For cell counting, 400X bright-field images of the granule cell layer of the dentate gyrus, the *stratum pyramidale* of the CA1, and the cortical layer III of the parietal association cortex were acquired using an inverted microscope (Zeiss® Axio Observer 7). The approximate locations of the captured regions are shown in Fig. 4A. Total cells within the neuronal layer were counted using ImageJ software in a defined region of interest (ROI) of 100 × 100µm.

### Immunofluorescence

Brain sections were deparaffinized in xylene, rehydrated through decreasing ethanol concentration series, and antigen retrieval was performed by heating the slides at 115 °C for 15 min in 10 mM citrate buffer with 0.05% polysorbate 20, pH 6.0. The slides were blocked with 3% NGS in PBS with 0.3% Triton™ X-100 (ThermoFisher® #85,113) for 40 min at 4 °C; and then incubated with the primary antibodies (diluted in blocking solution) overnight at 4 °C. The next day, the slides were incubated with the specific secondary antibodies diluted in PBS with 0.3% Triton™ X-100 and DAPI (1:1000; Invitrogen® #D3571) at room temperature for 2 h in the dark. All primary and secondary antibodies used are listed in Table 1. The slides were coverslipped with antifading mounting medium (ProLong™—ThermoFisher® #P36934). Images of the dentate gyrus, CA1 and cerebral cortex were acquired at 400X magnification using an inverted microscope (Zeiss® MODEL). Mean fluorescence intensities (MFI) were calculated in ImageJ software (Schneider et al. 2012).

**Table 1:** Primary and secondary antibodies used in immunofluorescence labeling

Marker	Brand	Code	RRID	Dilution
Primary antibodies				
GFAP	Invitrogen®	MA5-12023	AB_10984338	1:500
Iba-1	Cell Signaling®	17198	AB_2820254	1:100
Secondary antibodies				
Anti-Mouse + AlexaFluor™ 555	Invitrogen®	A21127	AB_2535769	1:500
Anti-Rabbit + AlexaFluor™ 488	Invitrogen®	A11008	AB_143165	1:500

### Brain water content

Animals at PND8 were euthanized as described in the “Histological Processing” section but perfused only with 0.9% saline (1 mL/g) and not fixed. Fresh brain tissue was immediately weighed to obtain the wet weight and then dried at 80 °C for 24 h. After drying, tissues were weighed again to obtain the dry weight. Brain water content was calculated as: Brain water content (%) = ((wet weight—dry weight)/wet weight) × 100 (Hu et al. 2020).

### Behavioral assessment

All behavioral tests were performed in the afternoon (between 1 p.m. and 6 p.m.). The same researchers conducted all assessments and were blinded to the experimental groups during both behavioral testing and evaluation.

At PND8, the negative geotaxis test was used to evaluate early sensorimotor function. Each pup was placed facing downward on a 40° inclined surface, and the latency for the animal to complete a 180° turn was measured manually, with a maximum cutoff time of 60 s (Feather-Schussler and Ferguson 2016). An additional trial was conducted if the animal fell from the apparatus, and in case of a second fall, the maximum time was assigned.

At PND14, the olfactory discrimination test was performed to assess early cognitive and sensorimotor function. The apparatus consisted of a box (40 × 30 × 18 cm) covered with clean bedding, except for a 10 × 30 cm area on one side of the floor which was covered with home bedding (with the dam’s and littermates’ scent). Each pup was placed on the clean bedding area, facing the home bedding area, and recorded from above for 2 min. Video recordings were analyzed using the ANY-maze software (v.7.4) and the following parameters were measured automatically: time spent in the home bedding area, latency to first reach the home bedding, and total number of crossings between areas.

At PND20, the cylinder test was conducted to evaluate motor function based on forepaw use (ipsilateral or contralateral to carotid occlusion). The animal was placed inside a glass beaker (13 cm in diameter and 19 cm in height) and video recorded from below for 5 min. During rearing, the number of touches with the right and left forepaws against the cylinder wall was manually counted. Animals with brain injury due to HI are expected to show impaired use of the left forepaw (contralateral to the injured hemisphere), leading to asymmetric use of the forepaws. The percentage of left forepaw use was calculated as: Left forepaw use (%) = (left forepaw touches/total forepaw touches) × 100.

### Developmental milestones

Animals were weighed daily from PND7 to PND21. Body weights were plotted as a growth curve, and the area under the curve (AUC) was calculated as an indicator of overall body weight gain during the period. The AUC was calculated using GraphPad Prism® software, considering the initial weight as the baseline and treating weights below the baseline as negative values. The day of upper and lower incisor eruption was recorded, as well as the day of eye opening for each eye separately, defined as the first observation of complete eyelid opening.

### Statistical analysis

Each sex was evaluated separately, yet no direct comparisons between them were performed. All statistical analyses were performed using GraphPad Prism® software (version 8.0.2). Outliers were identified using the ROUT method (Q = 1%), and data normality was assessed using the Shapiro–Wilk test. Data with a normal distribution were presented as mean ± SEM and were evaluated by one-way ANOVA followed by Tukey or by Welch’s ANOVA followed by Games-Howell when variances differed between groups. Data without a normal distribution were presented as median and interquartile range and were analyzed using the Kruskal–Wallis test. In the case of the right and left eye-opening comparison, the Wilcoxon test was used. The tests used for each dataset are specified in the corresponding figure captions. Statistical significance was set as *p* ≤ 0.05.

## Results

### Mortality and body temperature

A total of 19 rat pups died (7 during surgery, 7 during hypoxia and 5 on the days following HI). Since deaths during surgery were caused by carotid rupture or failure to recover from anesthesia, which represent methodological limitations rather than hypoxia resistance, they were not accounted for in the mortality calculation. Of the 7 animals that died during hypoxia, 4 were males and 3 were females. Of the 5 animals that died during the days following HI, 2 were females from the HI group and 2 were females from the HI + TH group, and the remaining animal was a SHAM male that did not gain weight and was not considered in the mortality calculation. Thus, considering a total of 121 animals submitted to HI, we obtained an overall mortality of 9.1%. Considering 62 males submitted to HI, a mortality of 6.5% was observed and in the 59 females a mortality of 11.9%.

The body temperature of rat pups during normothermia (36°C) or hypothermia (32°C), as well as the measurements with the infrared and rectal thermometers in the sentinel animals are shown in Fig. 1B. We observed that the mean infrared temperatures in the hypothermic animals were consistently higher than the mean rectal temperature by 0.5–1 °C.

### Brain injury

**Fig. 2:**
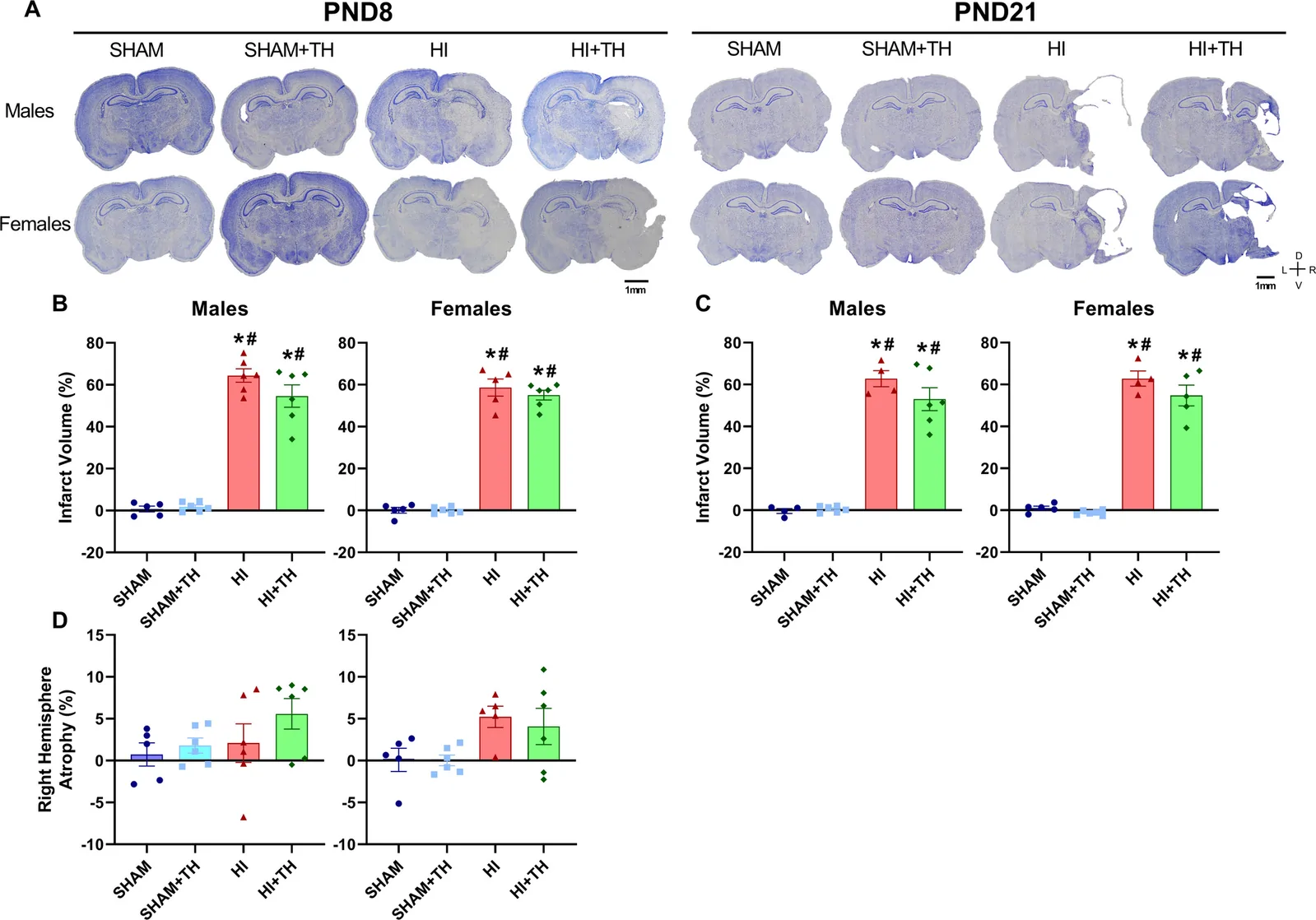
Volume of brain injury in Nissl-stained coronal sections of neonatal male and female rat pups submitted to hypoxia–ischemia. (**A**) Representative images of sections containing the hippocampus are shown for PND8 and PND21. Injured tissue can be observed in HI and HI + TH animals' right hemispheres as pale regions at PND8 or as areas lacking tissue at PND21. Infarct volume was measured as a percentage of the contralateral hemisphere volume in PND8 (**B**) and in PND21 (**C**) for both sexes. The right hemisphere atrophy at PND8 was also measured (**D**). Results are shown as mean ± SEM and were compared by Welch’s ANOVA followed by Games-Howell. *p < 0.05 vs. SHAM; ^#^*p* < 0.05 vs. SHAM + TH. D: dorsal; HI: hypoxia–ischemia; L: left; R: right; TH: therapeutic hypothermia; V: ventral

A significant brain injury volume was observed in the right hemisphere (ipsilateral to the carotid occlusion) of males and females submitted to HI when compared to controls (Fig. 2). The lesion volume was approximately 60% in most animals of HI and HI + TH groups, considered a moderate to severe injury. Both groups exhibited significant brain injury in males and females at PND8 (males: F_(3, 9.49)_ = 130.9, *p* < 0.001; females: F_(3, 8.26)_ = 204.8, *p* < 0.001) and PND21 (males: F_(3, 6.83)_ = 97.38, *p* < 0.001; females: F_(3, 6.79)_ = 118.5, *p* < 0.001). Although the affected area size was similar, the injury at PND8 was observed as a pale region with shrunken cells and empty spaces, whereas at PND21 we observed complete atrophy and lack of tissue. When atrophy alone was considered at PND8 (total hemisphere volumes, regardless of tissue health), no significant effects of HI or TH were found.

**Fig. 3:**
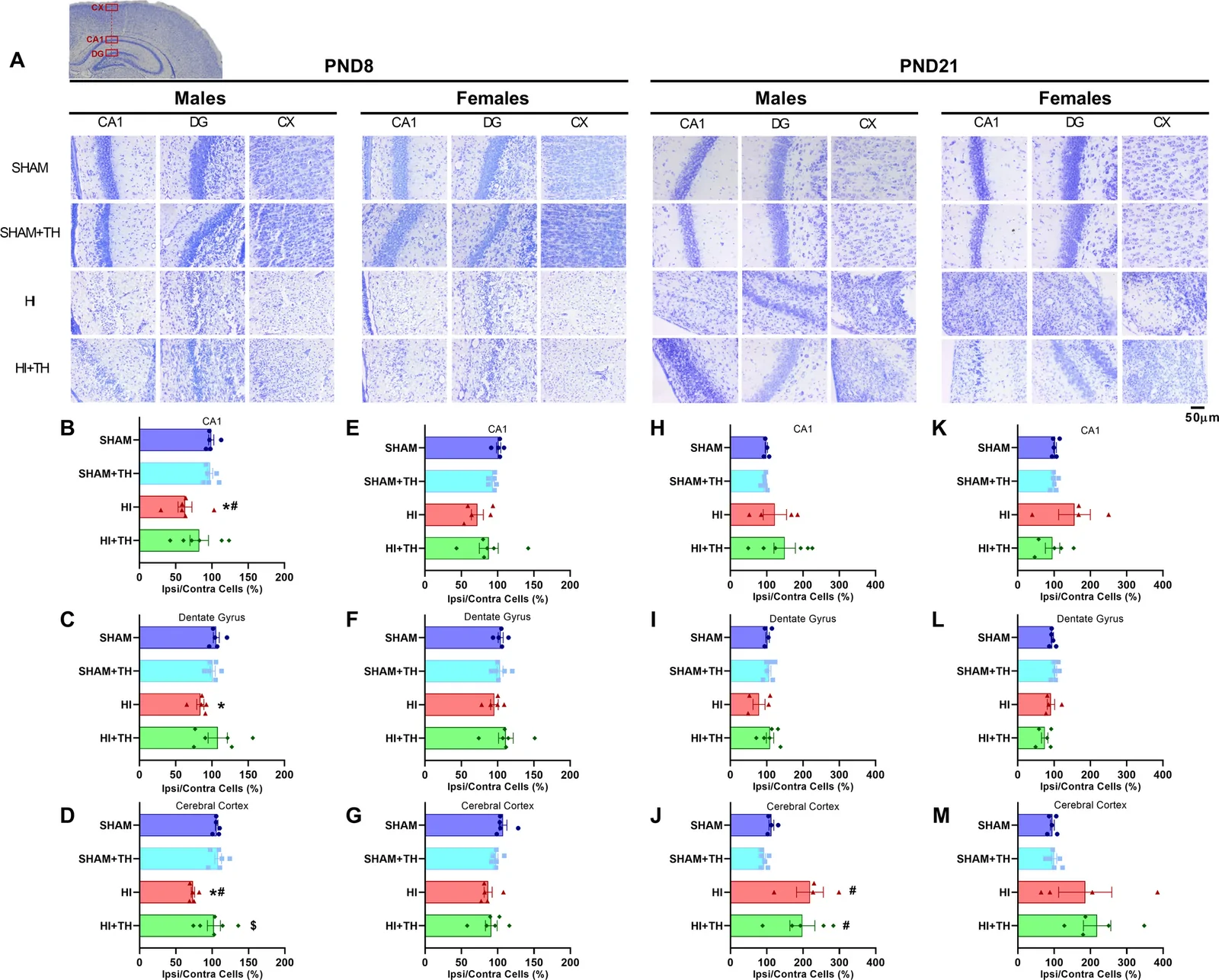
Cell counting performed at PND8 and PND21 in Nissl-stained coronal sections of male and female rat pups submitted to hypoxia–ischemia. (**A**) Representative images of the right (ipsilateral) brain hemisphere regions analyzed in the CA1, dentate gyrus and cerebral cortex. For each experimental group of each age, the representative images of the different regions were acquired from the same animal. A 100 × 100µm ROI was positioned in the *stratum pyramidale* of the CA1, the granule cell layer of the dentate gyrus or the cortical layer III of the parietal association cortex, and all cells in these layers were counted indiscriminately. The ratio of cells counted in the right (ipsilateral) and the left (contralateral) hemispheres are shown in PND8 males (**B**, **C** and **D**) and females (**E**, **F** and **G**), and for PND21 males (**H**, **I** and **J**) and females (**K**, **L** and **M**). Results are shown as mean ± SEM and were compared by one-way ANOVA followed by Tukey. The DG of PND8 males (**C**), the CA1 and DG of PND21 males (**H** and **I**) and the CX of PND21 females (**M**) were compared with Welch’s ANOVA followed by Games-Howell. *p < 0.05 vs. SHAM; ^#^p < 0.05 vs. SHAM + TH; ^$^*p* < 0.05 vs. HI + TH. CX: cerebral cortex; DG: dentate gyrus, HI: hypoxia–ischemia, TH: therapeutic hypothermia

When the total number of cells was quantified (Fig. 3), we observed that males at PND8 exhibited reduced cell counts in the CA1 (F_(3, 19)_ = 3.80, *p* = 0.027), the DG (F_(3, 9.58)_ = 3.84, *p* = 0.048) and the cerebral cortex (F_(3, 18)_ = 7.59, *p* = 0.002). In all these areas, TH treatment was able to reduce cell loss. On the other hand, females did not differ in cell counts between groups. At PND21, males showed an increase in the number of cells in the cerebral cortex of animals submitted to HI (F_(3, 15)_ = 6.83, *p* = 0.004), whereas in CA1 and DG no significant differences were observed. In females at PND21 the only area affected was the DG, which showed reduced cell counts in the HI + TH groups (F_(3, 16)_ = 3.96, *p* = 0.028). Representative images of the contralateral hemisphere are shown in Fig. S1.

### Cerebral edema

**Fig. 4:**
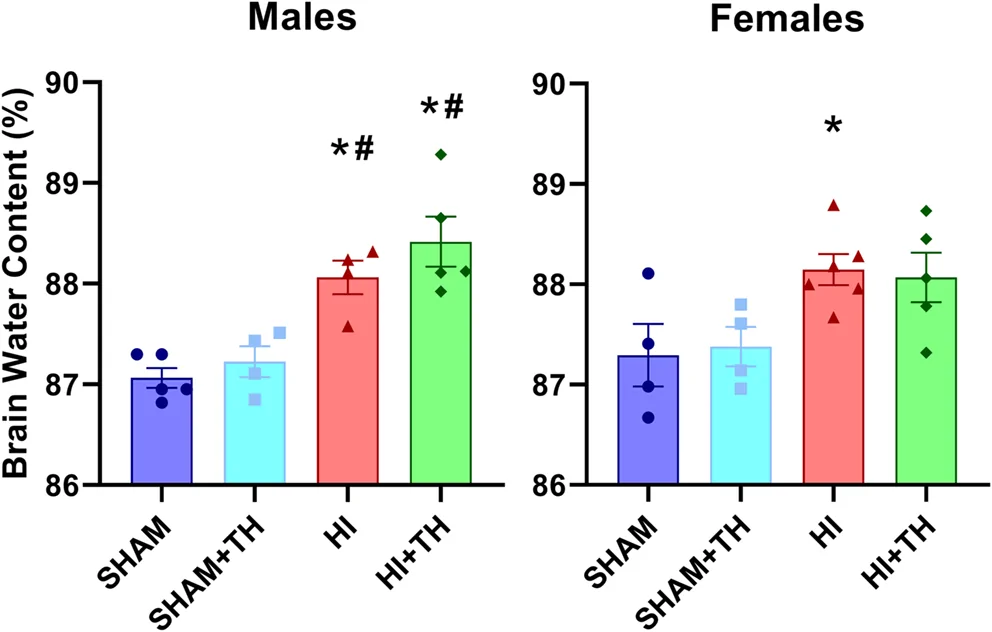
Brain water content was measured as the ratio between wet and dry brain weights at PND8 in male and female rats after HI as an indicator of cerebral edema. Results are presented as mean ± SEM and were compared by one-way ANOVA followed by Tukey. *p < 0.05 vs. SHAM; ^#^*p* < 0.05 vs. SHAM + TH. HI: hypoxia–ischemia; TH: therapeutic hypothermia

We evaluated brain water content as an indicator of cerebral edema (Fig. 4). In males, we observed increased brain water content in animals submitted to HI, regardless of TH treatment (F_(3, 14)_ = 13.74, *p* < 0.001). In females, the HI-induced increase in brain water content was ameliorated by TH (F_(3, 15)_ = 3.95, *p* = 0.029).

### Microglial reactivity

**Fig. 5:**
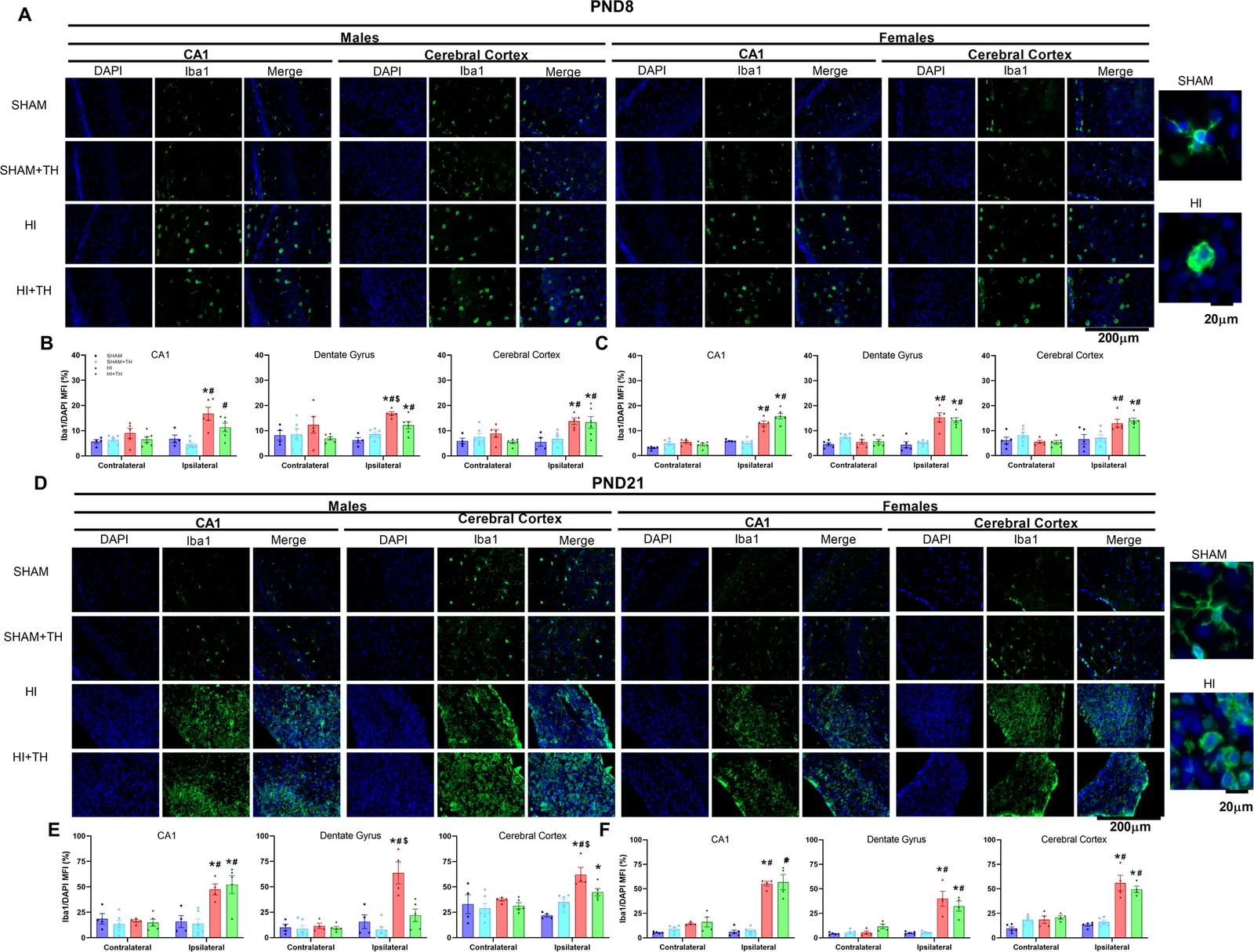
Microglial reactivity measured as Iba1 MFI at PND8 or PND21 in male and female pups submitted to hypoxia–ischemia. Representative images (**A** and **D**) of the right (ipsilateral) brain hemisphere regions analyzed in CA1, dentate gyrus and cerebral cortex. Microglia from HI and HI + TH animals showed an amoeboid morphology rather than a ramified morphology, as shown in detail in the right. For each experimental group of each age, the representative images of the different regions were acquired from the same animal. Iba1 MFI (green), in relation to DAPI MFI (blue), is shown at PND8 in males (**B**) and females (**C**) and at PND21 in males (**E**) and females (**F**). Results are presented as mean ± SEM and were compared by one-way ANOVA followed by Tukey. *p < 0.05 vs. SHAM; ^#^*p* < 0.05 vs. SHAM + TH; ^$^*p* < 0.05 vs. HI + TH. HI: hypoxia–ischemia, MFI: mean fluorescence intensity; TH: therapeutic hypothermia

Microglial reactivity measured by Iba1 MFI was increased in all regions of the ipsilateral brain of HI animals from both sexes (Fig. 5) both at PND8 (males CA1: F_(3, 17)_ = 9.50, *p* < 0.001; males DG: F_(3, 17)_ = 16.30, *p* < 0.001; males CX: F_(3, 17)_ = 5.97, *p* = 0.006; females CA1: F_(3, 18)_ = 39.95, *p* < 0.001; females DG: F_(3, 18)_ = 23.62, *p* < 0.001; females CX: F_(3, 18)_ = 7.46, *p* = 0.002) and at PND21 (males CA1: F_(3, 15)_ = 10.18, *p* < 0.001; males DG: F_(3, 15)_ = 13.46, *p* < 0.001; males CX: F_(3, 15)_ = 15.79, *p* < 0.001; females CA1: F_(3, 14)_ = 54.62, *p* < 0.001; females DG: F_(3, 14)_ = 19.89, *p* < 0.001; females CX: F_(3, 14)_ = 31.03, *p* < 0.001). Males from the HI group showed greater Iba1 MFI than females, particularly in the dentate gyrus in PND21. Interestingly, at PND8, TH was able to partially reduce Iba1 MFI in the CA1 and the dentate gyrus of males, but in none of the regions in females. At PND21, TH prevented the increase in Iba1 MFI in the dentate gyrus and cerebral cortex of males, and in none of the regions in females. In both ages and sexes, HI and HI + TH microglia in the ipsilateral hemisphere had an ameboid morphology typical of reactive microglia, in contrast to the ramified microglia, typical of the surveillance state, found in the SHAM and SHAM + TH animals. In the contralateral hemisphere, HI did not increase Iba1 MFI in any of the regions. Microglia in the contralateral hemisphere also exhibited a ramified (surveillance state) morphology in all groups (Fig. S2).

### Astrocytic reactivity

**Fig. 6:**
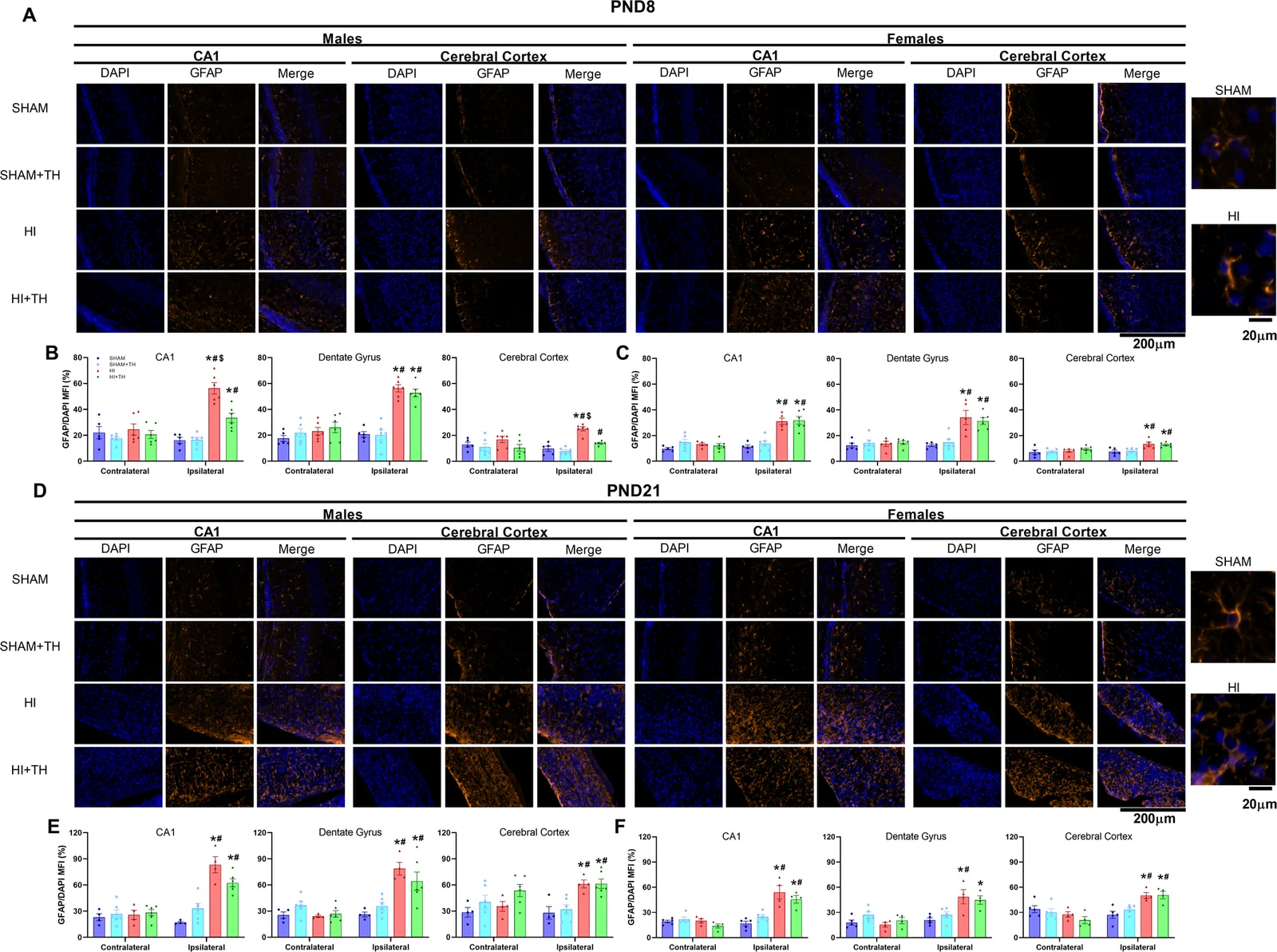
Astrocytic reactivity measured as GFAP MFI at PND8 or PND21 in male and female pups submitted to hypoxia–ischemia. Representative images (**A** and **D**) of the right (ipsilateral) brain hemisphere regions analyzed in CA1, dentate gyrus and cerebral cortex. Astrocytes from HI and HI + TH animals showed thickened branches and reactive morphology, as shown in detail in the right. For each experimental group of each age, the representative images of the different regions were acquired from the same animal. GFAP MFI (orange), in relation to DAPI MFI (blue), is shown at PND8 in males (**B**) and females (**C**) and at PND21 in males (**E**) and females (**F**). Results are presented as mean ± SEM and were compared by one-way ANOVA followed by Tukey. *p < 0.05 vs. SHAM; ^#^*p* < 0.05 vs. SHAM + TH; ^$^*p* < 0.05 vs. HI + TH. HI: hypoxia–ischemia, MFI: mean fluorescence intensity; TH: therapeutic hypothermia

Astrocytic reactivity measured by GFAP MFI was also increased in all evaluated regions of the ipsilateral hemisphere of HI animals from both sexes (Fig. 6) both at PND8 (males CA1: F_(3, 19)_ = 33.01, *p* < 0.001; males DG: F_(3, 19)_ = 40.45, *p* < 0.001; males CX: F_(3, 19)_ = 33.87, *p* < 0.001; females CA1: F_(3, 18)_ = 20.52, *p* < 0.001; females DG: F_(3, 18)_ = 11.96, *p* < 0.001; females CX: F_(3, 18)_ = 7.80, *p* = 0.002) and at PND21 (males CA1: F_(3, 16)_ = 22.99, *p* < 0.001; males DG: F_(3, 16)_ = 9.35, *p* < 0.001; males CX: F_(3, 16)_ = 9.73, *p* < 0.001; females CA1: F_(3, 14)_ = 14.36, *p* < 0.001; females DG: F_(3, 14)_ = 7.14, *p* = 0.004; females CX: F_(3, 14)_ = 10.19, *p* < 0.001). Males from the HI group also showed higher GFAP MFI levels than females in all regions at both ages. In males, TH partially reduced GFAP MFI increase in the CA1 and cerebral cortex at PND8, whereas in females of this age TH did not reduce GFAP MFI increase in any region. At PND21, TH did not significantly reduce the increase in GFAP MFI in any of the regions in both sexes. At both ages and in both sexes, HI and HI + TH astrocytes in the ipsilateral hemisphere had a reactive-like morphology, displaying thicker branches and swollen soma, in contrast to the typical morphology found in the SHAM and SHAM + TH animals. In the contralateral hemisphere, HI did not increase GFAP MFI in any of the regions. Astrocytes in the contralateral hemisphere presented a healthy morphology in all groups (Fig. S3).

### Behavioral parameters

**Fig. 7:**
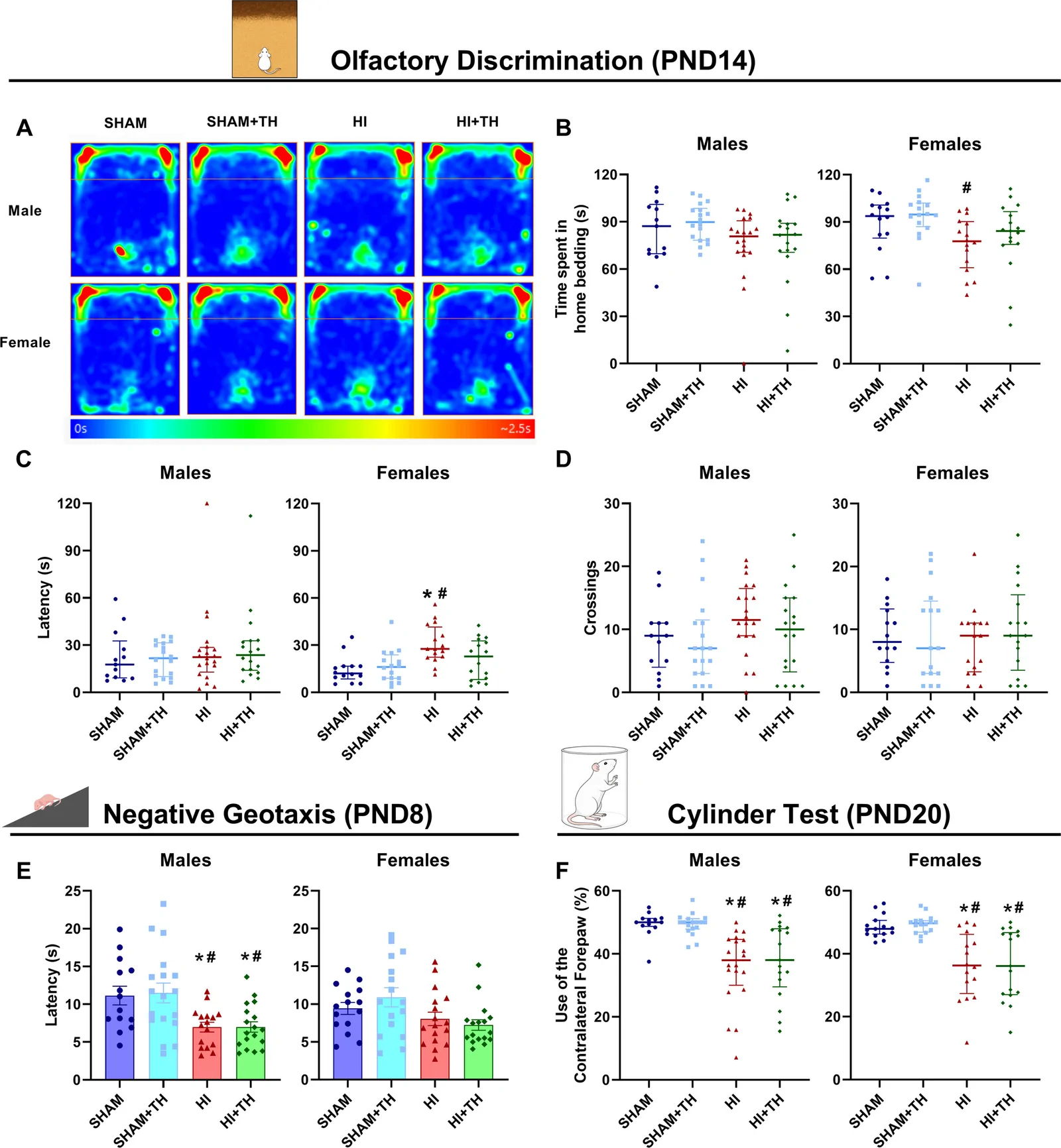
Behavioral Parameters. (**A**) Representative top view of the box used in the olfactory discrimination test. The smaller area (above the line) contains home bedding, and the larger area contains clean bedding. Heat maps show the mean time spent in each area for each experimental group (total 2 min) per experimental group and sex. (**B**) Total time spent in the home bedding in the olfactory discrimination test. (**C**) Latency to first reach the home bedding in the olfactory discrimination test. (**D**) Number of crossings through the imaginary line that divides the two bedding types, representing locomotor activity. (**E**) Latency to make a 180° turn in the negative geotaxis test in males and females. (**F**) Percentage of use of the contralateral forepaw in the cylinder test. Results of the negative geotaxis are presented as mean ± SEM, the remaining as median and interquartile range. Comparisons in the negative geotaxis were conducted by Welch’s ANOVA followed by Games-Howell, the remaining by Kruskal–Wallis followed by Dunn. *p < 0.05 vs. SHAM; ^#^*p* < 0.05 vs. SHAM + TH. HI: hypoxia–ischemia, TH: therapeutic hypothermia

Latency in the negative geotaxis test at PND8 was reduced in males from the HI or HI + TH groups (F_(3, 32.45)_ = 5.78, *p* = 0.003), and in females only in the HI + TH group (F_(3, 31.82)_ = 4.64, *p* = 0.008) as compared to the SHAM groups (Fig. 7E). Four males from the HI group and one female from the HI + TH group fell twice during the test and were excluded since they were identified as outliers. Olfactory discrimination performance at PND14 was impaired by HI only in females, observed as a reduction in time spent in the home bedding area (Fig. 7B) (K = 10.78, *p* = 0.013, n = 63) and an increase in latency to reach the home bedding (Fig. 7C) (K = 14.27, *p* = 0.003, n = 63). The number of crossings was not different between the groups in any sex, indicating there were no locomotor deficits (Fig. 7D) (males: K = 4.10, *p* = 0.251, n = 69; females: K = 0.48, *p* = 0.924, n = 63). In the cylinder test at PND20, we observed motor deficits caused by HI in both sexes, and none were reverted by TH (Fig. 7F) (males: K = 31.93, *p* < 0.001, n = 68; females: K = 25.64, *p* < 0.001, n = 64). One male from the HI + TH group touched the walls of the cylinder less than 10 times and was excluded from the analysis.

### Developmental parameters

**Fig. 8:**
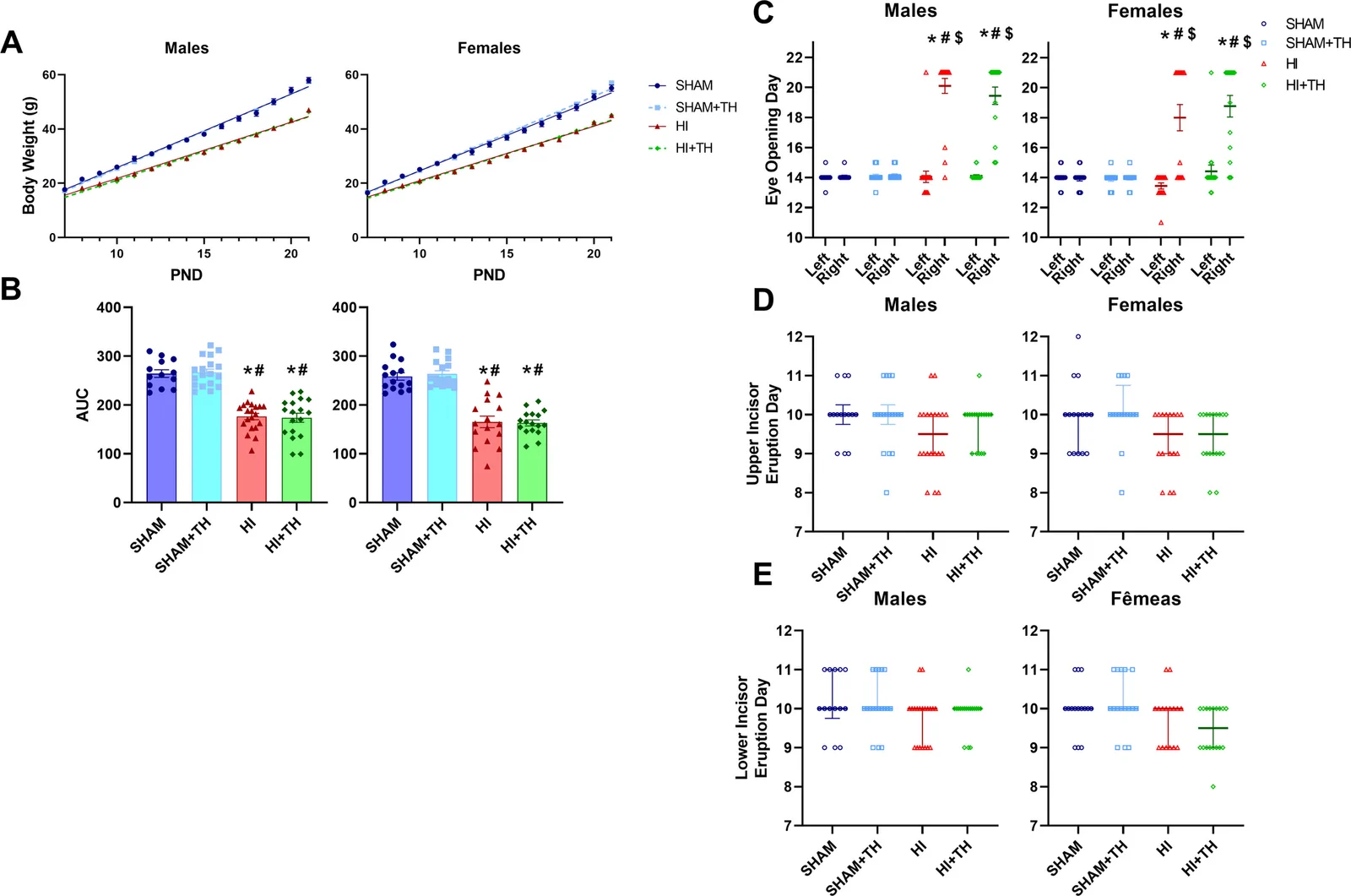
Developmental parameters measured in rat pups of both sexes after neonatal HI exposure. (**A**) Body weight curves of both sexes and their respective AUC (**B**), representative of body weight gain. (**C**) The day of eye opening in males and females for the right and left eyes, defined as the first observation of total eyelid opening. (**D** and **E**) Day of the first observation of the eruption of upper and lower incisors. AUCs are shown as mean ± SEM and were analyzed by one-way ANOVA followed by Tukey. The remaining results are presented as median and interquartile range and were compared by Kruskal–Wallis followed by Dunn. For comparisons between the right and the left eye within each group, the Wilcoxon test was used. *p < 0.05 vs. SHAM; ^#^*p* < 0.05 vs. SHAM + TH; ^$^*p* < 0.05 vs. left eye. AUC: area under the curve, HI: hypoxia–ischemia, TH: therapeutic hypothermia

Body weight was reduced from PND7 to PND21 in animals of both sexes submitted to HI when compared to SHAM, an effect that was not reverted by TH (Fig. 8A-B) (males: F_(3, 65)_ = 44.97, *p* < 0.001; females: F_(3, 60)_ = 44.77, *p* < 0.001). A delay in the opening of the right eye (ipsilateral to carotid occlusion) compared to the left eye was observed in the HI and HI + TH groups of both sexes (Fig. 8C) (HI males: W = 190.0, *p* < 0.001, n = 20; HI + TH males: W = 171.0, *p* < 0.001, n = 18; HI females: W = 105.0, *p* < 0.001, n = 16; HI + TH females: W = 105.0, *p* < 0.001, n = 17). When comparisons were made between experimental groups, the delay in the opening of the right eye was still observed in the HI and HI + TH groups of both sexes (males: K = 54.10, *p* < 0.001, n = 69; females: K = 31.04, *p* < 0.001, n = 64). Females from the SHAM + TH group exhibited delayed upper incisor eruption when compared with the HI group, and in lower incisor eruption when compared to HI + TH group (upper: K = 10.17, *p* = 0.017, n = 65; lower: K = 8.65, *p* = 0.035, n = 65). Upper and lower incisor eruption in males were not affected by HI or TH (Fig. 8D-E) (upper: K = 5.27, *p* = 0.1531, n = 70; lower: K = 4.34, *p* = 0.227, n = 70).

## Discussion

As TH remains the only clinical treatment available for neonatal HI, understanding the underlying mechanisms related to its limited efficacy is essential for optimizing TH protocols and developing alternative or complementary therapies. Chronic neuroinflammation is a major cause of long-term brain injury after HI, yet it remains poorly investigated in the context of TH. In this study, we evaluated the effects of TH on astrocytic and microglial reactivity at two distinct time points after neonatal HI (PND8 and PND21), representing early and later stages of injury progression. To the best of our knowledge, this is the first study to evaluate these parameters at these time points after TH while also considering sex-specific differences.

In experimental TH protocols, body temperature is commonly monitored using a rectal probe in a restrained sentinel animal, which must be excluded from subsequent analysis since restraint-induced stress may itself exert neuroprotective effects (Gundersen et al. 2023). Here, body temperature during hypothermia was monitored using an unrestrained sentinel animal with both rectal and infrared temperature recordings. This approach was chosen to maintain conditions as similar as possible to those experienced by the remaining pups, ensuring comparable heat exchange with the hypothermic environment. The flexible rectal probe was safely secured to the tail, and no rectal injuries were observed. Infrared temperature readings were consistently 0.5–1°C higher than rectal values, suggesting that infrared monitoring may provide a reliable, non-invasive method for temperature control that is applicable for all animals. Similar consistency has been observed in normothermic and ethanol-induced hypothermic adult mice (Saegusa and Tabata 2003). This approach may eliminate the need for a sentinel animal (reducing the number of animals required) and allow individual temperature monitoring in all pups.

To evaluate the neuroprotective effects of TH on injury severity and progression, brain injury volume was assessed. We found that a prolonged hypoxic exposure (90 min duration), caused severe brain injury, as reflected by a loss of around 60% of brain tissue (Wood et al. 2016). Although injury volume was similar at early and later time points (PND8 and PND21, respectively) the lesion progressed from tissue undergoing active cell death to a marked atrophy of the affected region. This difference is supported by the lack of significant atrophy at PND8. TH failed to reduce brain injury volume at either age, which is consistent with the limited efficacy of hypothermia in severe injury paradigms (Sabir et al. 2012; Wood et al. 2016). Accordingly, using a PND10 HI model, Patel et al. (2015) showed that TH is not protective 24 h after injury, although its protective effects were seen 2 and 12 weeks later. Moreover, studies using milder hypoxic insults in neonatal rats have shown that TH can significantly reduce brain injury, highlighting the strong influence of injury severity on therapeutic outcome. In our previous studies using PND7 pups, a hypoxia duration of 75 min (Zang et al. 2023) or 90 min (Spies et al. 2025) produced a moderate brain injury (~ 40% of lesion) that was reduced by TH. Studies with PND11 pups (older and more susceptible to hypoxia) also showed that at 60 min hypoxia exposure produced a moderate damage which was reduced by TH (Fabres et al. 2025).

Beyond macroscopic injury, we evaluated cell loss through cell counting and observed that TH prevented the loss in all evaluated brain regions in males at the early time point (PND8). We have previously shown that HI reduces cell counting and increases cell degeneration in the hippocampus at PND9, which had been also prevented by TH (Zang et al. 2023). Neuron nuclei become pyknotic following HI and it has been reported that TH can preserve nuclei morphology (Shah et al. 2021), but this neuroprotective effect was not seen here. Interestingly, despite a clear brain injury volume observed in females, cell counting was not significantly reduced following HI. This finding may reflect sex-specific neuroprotective mechanisms that limit cell death in preserved brain regions in females.

We also observed an increased number of cells in the cerebral cortex at PND21 in males. It should be noted that, although Nissl staining preferentially labels neurons, this distinction becomes unreliable in injured tissue, where many cells exhibit condensed apoptotic nuclei. For this reason, all cells within the selected regions were counted regardless of cell type. In this context, an increased number of cells may therefore reflect astrocytic proliferation, microglia migration (both processes observed here), as well as migration of other immune cells to the injured area, masking possible neuronal death in all regions evaluated. In fact, Fabres et al. (2025) showed that two months after neonatal HI, a decrease in NeuN^+^ neuron counting is seen in the hippocampal CA1 area ipsilateral to carotid artery occlusion, but it is accompanied by an increase in the number of GFAP^+^ and Iba1^+^ cells. An additional explanation for the increase in cell number is enhanced neurogenesis in the subventricular zone and migration of neuroblasts toward the injured cortex (Plane et al. 2004; Yang et al. 2007), a regenerative mechanism characteristic of the neonatal brain.

Cerebral edema is another hallmark of early brain injury (Wagner et al. 2002) and was assessed here by measuring brain water content. In our previous work, we had qualitatively observed the presence of cerebral edema at PND9; however, brain wet weight had been reduced by HI and this reduction had not been prevented by TH. Considering that HI markedly reduces brain mass, tissue loss may have confounded the interpretation of edema and masked potential effects of TH (Nunes et al. 2024). In the present study, we demonstrated that neonatal HI increases brain water content, thereby confirming the presence of cerebral edema. Increased BBB permeability, a process closely related to neuroinflammation, is a major contributor to edema. Peripheral immune cell infiltration following BBB disruption can further amplify neuroinflammation. In a previous meta-analysis, we had shown that males present increased astrocytic and microglial reactivity, as well as increased infiltration of peripheral immune cells following neonatal HI (Nunes et al. 2025). Thus, sex-dependent vulnerability to neuroinflammation may contribute to differential barrier integrity, potentially explaining why, in the present study, TH reduced brain water content only in females. Enhanced clearance of interstitial fluid via glymphatic and meningeal lymphatic pathways stimulated by TH may also contribute to edema reduction, as recently shown in an intraventricular hemorrhage model (Yang et al. 2026)—although the role of TH in modulating these systems after neonatal HI remains unclear and previous studies using traumatic brain injury models suggest that TH has the opposite effect (Bai et al. 2022; Gu et al. 2023). Impairments in glymphatic function were described for neonatal HI (Shen et al. 2025), but the effects of subsequent TH treatment have not been investigated in this model. Additionally, it has been shown that peripheral immune cells can be found in brain parenchyma up to a year after HI (Bolini et al. 2024). Thus, further investigation of the BBB and glymphatic function, particularly at later stages, is warranted.

Neuroinflammatory responses following HI are initiated by rapid activation of microglia, the resident immune cells of the central nervous system (Jithoo et al. 2024). Depending on injury stage and activation state, microglia may play either pro- or anti-inflammatory roles, through cytokine release, antigen presentation to infiltrated T cells, and phagocytosis of cellular debris. Tsuji et al. (2020) demonstrated worse outcomes in microglia-depleted mice, suggesting that microglia possess a more neuroprotective than detrimental balance. Here, microglia from animals submitted to HI presented ameboid morphology, indicating reactivity, and an increased Iba1 MFI was seen in all ages, which was more pronounced in males at PND21. Although we did not assess microglial polarization, we suggest a detrimental role for microglia, since males tend to show greater microglial activation and leukocyte infiltration than females in early stages of HI, accompanied by greater injury (Al Mamun et al. 2018; Mirza et al. 2015).

Astrocytes also play a central role in neuroinflammation. Following stress or injury, astrocytes proliferate and undergo morphological changes characterized by thickened branches, a process known as astrogliosis (De Fraga et al. 2021), a response that can persist for long after the initial insult (Diaz et al. 2017). Reactive astrocytes are responsible for the formation of glial scars and produce diverse pro-inflammatory cytokines, metalloproteinases, and reactive oxygen species, thereby exacerbating neuroinflammation and BBB disruption (Li et al. 2025). Here, male pups that had undergone HI presented greater GFAP MFI (suggesting greater astrocytic reactivity) than females at PND8 and PND21. This pattern was more pronounced than that observed for microglia.

Both microglial and astrocytic reactivity were partially reduced by TH at PND8 in some brain regions of males, whereas at PND21 only microglial reactivity was reduced. Although TH has been shown to reduce glial activation in various injury models, evidence suggests that its anti-inflammatory effects are largely transient. For example, TH was shown to reduce microglial activation and migration in vitro and in vivo in a stab injury model (Seo et al. 2012). Reduction in Iba1^+^ cells in the injured brain after neonatal HI and TH treatment has also been described (Shah et al. 2021). Some studies have shown that astrogliosis is reduced by TH up to one week after neonatal HI (Fabres et al. 2022; Xiong et al. 2009). The study by Goffigan-Holmes et al. (2019) showed that TH tended to reduce GFAP immunoreactivity early, but not in later stages, which is in agreement with our results. Our findings support this notion, as reactive glial morphology persisted despite early reductions in fluorescence intensity, potentially contributing to a later resurgence of neuroinflammation.

While histological and inflammatory markers provide important insights into brain injury, functional consequences are best evaluated through behavioral assessments. Negative geotaxis is commonly used to evaluate sensorimotor function in neonatal HI models. Surprisingly, HI resulted in decreased latency in this test, a finding consistent with our previous observations (Zang et al. 2023). Similar responses (faster turning) in PND16 animals have been attributed to increased anxiety-like behavior and reduced exploratory drive (Diaz et al. 2017). Aligned to this hypothesis, it is well known that hippocampal lesions in adult rodents produce a marked hyperactivity behavior (Thompson et al. 2018). Given the high injury severity and the vulnerability of the hippocampus to hypoxic damage, this altered behavioral pattern may reflect maladaptive hyperactivity rather than preserved function.

At later stages, the cylinder test revealed clear motor impairments, consistent with its higher sensitivity and ability to capture injury laterality. Behavioral deficits may become more homogeneous over time as injury progression stabilizes. Impairments in the cylinder test were observed in earlier periods, as PND20 (Tassinari et al. 2020) and PND24 (Tassinari et al. 2024), although HI did not affect the performance in this test later, at PND45 (Sanches et al. 2017), likely reflecting enhanced neonatal brain plasticity. In fact, it has been reported that some behavioral deficits tend to be resolved with time (Barth and Stanfield 1990; Felt et al. 2003). In the context of TH, Lee et al. (2010) demonstrated ameliorated forepaw use asymmetry in PND35 males. On the contrary, we found that TH was not effective in preventing motor deficits in this paradigm. This lack of efficacy may be related to both injury severity and differences in the timing of behavioral assessment.

In addition to motor performance, we evaluated olfactory discrimination as a potential early indicator of cognitive dysfunction. This behavior relies on neural circuits involving the olfactory bulb, amygdala, and hippocampus (Sakano 2020), and is particularly relevant during early development. The maturation of odor-driven behaviors depends on odorant exposure from birth up to PND7, a moment known as olfactory critical period (Inoue et al. 2021). Thus, since odor-driven learning occurs before the induction of HI, impairments observed here likely reflect deficits in memory retrieval rather than acquisition. Female pups exposed to HI showed clear impairments in olfactory discrimination, spending less time in and taking longer to reach the home bedding. Interestingly, the impairment was observed only in females, which is consistent with previous suggestions that females may be more prone to cognitive dysfunction after HI (Netto et al. 2017). These effects were prevented by TH. Given that locomotor activity was not altered, motor deficits are unlikely to account for these findings. Although cognitive impairment provides a plausible explanation, deficits in olfactory sensory processing cannot be fully excluded.

Olfactory discrimination remains poorly investigated in neonatal HI; however, increased latency to reach the home bedding has been reported in studies (Sanches et al. 2017, 2012). Using alternative odor-related tasks, Chen et al. (2022) also demonstrated olfactory impairments at later developmental stages (PND28 to PND35) after HI exposure at PND7. In addition, females have been shown to benefit more from TH than males in cognitive paradigms such as the food protection test (Saadat et al. 2025). In our previous studies, we evaluated olfactory discrimination for the first time in this context but did not detect significant effects of either HI or TH (Nunes et al. 2024; Zang et al. 2023). Here, the results obtained may be related to the establishment of different criteria to extend the analysis of the test to cover variables beyond latency and bedding preference: we evaluated total time spent in each area to reduce the impacts of increased exploratory behavior that happens as the animal ages, although PND14 rats are still expected to prefer home-related cues (Goodwin and Yacko 2004). In addition, the home bedding was positioned directly in front of the animal to avoid potential confounding effects of injury laterality.

Developmental parameters provided additional insight into overall health status. HI impaired body weight gain and delayed ipsilateral eye opening, and TH was not protective. Body weight gain was reduced by HI, a well-documented effect usually attributed to motor deficits that impair pups’ breastfeeding ability (Diaz et al. 2017; Lubics et al. 2005; Nunes et al. 2024; Tassinari et al. 2024, 2020). The delay in the opening of the eye ipsilateral to carotid occlusion is another usual consequence of HI (Lubics et al. 2005; Nunes et al. 2024; Tassinari et al. 2020). Common carotid occlusion can cause ischemia in the eye perfused by the ophthalmic artery, a branch of the carotid (Chan et al. 2015; Huang et al. 2012). We also suggest that eye opening can be impaired by motor impacts in eyelid control. Eruption of upper or lower incisors was not affected by HI, which is in line with other studies (Lubics et al. 2005; Schuch et al. 2016), likely due to its close temporal proximity to the injury. Importantly, the inclusion of a sham-hypothermia group allowed us to exclude deleterious effects of TH itself under the present experimental conditions, particularly regarding developmental and behavioral parameters, as described in a preterm anoxia model by Matsuda et al. (2021), but none of these parameters were affected in this group here, probably because of the use of TH at a later age.

This study has some limitations. The use of a severe injury model limits the generalizability of the findings to milder forms of HI, where TH is known to be more effective. Future studies employing less severe hypoxic insults are necessary to determine whether chronic neuroinflammation persists despite effective neuroprotection. In addition, cell counting would benefit from neuron-specific markers to better distinguish neuronal loss from glial proliferation and immune cell infiltration, processes that could be masking cell death and the neuroprotective effects of TH, especially at PND21. Moreover, the evaluation of other aspects of brain injury, such as myelinization and oligodendrocyte survival, could give new insights into TH efficacy and the impacts of neuroinflammation. The lack of specific markers for glial activation states and inflammatory mediators also limits mechanistic interpretation.

Despite these limitations, our results demonstrate that TH partially reduces early microglial and astrocytic reactivity after neonatal HI in males, but this effect is not sustained at later stages for astrocytes. These transient anti-inflammatory effects may contribute to the limited neuroprotective efficacy of TH with respect to brain injury, behavioral outcomes, and developmental parameters. As TH use is now considered to be near optimal (Zhou et al. 2020), researchers should focus on potential adjunct therapies that produce long-term anti-inflammatory effects and are capable of modulating the microglial response. Nonetheless, it has been challenging to acquire additional protection when combining TH and promising therapies such as xenon, erythropoietin, and stem cell-based strategies (Sabir 2025; Zhou et al. 2020). Moreover, the pronounced sex-dependent responses observed reinforce the importance of including both sexes in experimental designs and analyzing them separately when evaluating neuroprotective strategies, imposing additional challenges.

## Supplementary Information

Below is the link to the electronic supplementary material.Supplementary file1 (PDF 816 KB)

## Data Availability

The data that support the findings of this study are available from the corresponding author upon reasonable request.

## Abbreviations

AUC: Area under curve
BBB: Blood-brain barrier
CCA: Common carotid artery
DAPI: 4′,6-Diamidino-2-phenylindole
GFAP: Glial fibrillary acidic protein
HI: Hypoxia-ischemia
Iba-1: Ionized calcium-binding adaptor molecule 1
IL1-β: Interleukyn-1 beta
IL-6: Interleukyn-6
MFI: Mean fluorescence intensity
PND: Post-natal day
TH: Therapeutic hypothermia
TNF-α: Tumor necrosis factor alpha
