# Supplementary material for: Sex-specific effects of therapeutic hypothermia on short- and long-term microglial and astrocytic reactivity after severe neonatal hypoxia–ischemia
Source: Metab Brain Dis. 2026 Aug 29;41(1):194. doi: 10.1007/s11011-026-01957-y (PMC13525959; doi:10.1007/s11011-026-01957-y)
Supplement: Supplementary file 1 [file 11011_2026_1957_MOESM1_ESM.pdf]

Sex-specific effects of therapeutic hypothermia on short- and long-term microglial and astrocytic reactivity after severe neonatal hypoxia-ischemia

Supplementary Material

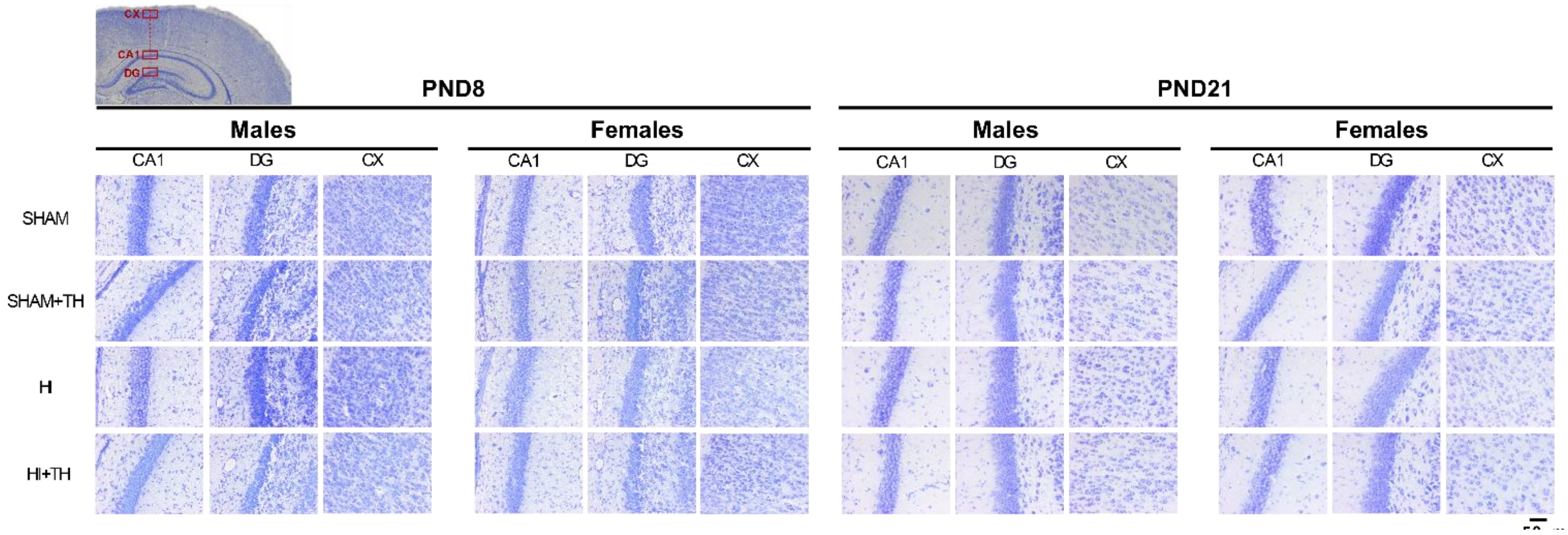

Fig. S1. Cell counting was performed at PND8 and PND21 in Nissl-stained coronal sections of male and female rat pups submitted to hypoxia-ischemia. Representative images of the left (contralateral) brain hemisphere regions analyzed in CA1, dentate gyrus and cerebral cortex are shown. A 100x100µm ROI was positioned in the *stratum pyramidale* of the CA1, the granule cell layer of the dentate gyrus or the cortical layer III of the parietal association cortex, and all cells in these layers were counted indiscriminately. For each experimental group of each age, the representative images of the different regions were acquired from the same animal.

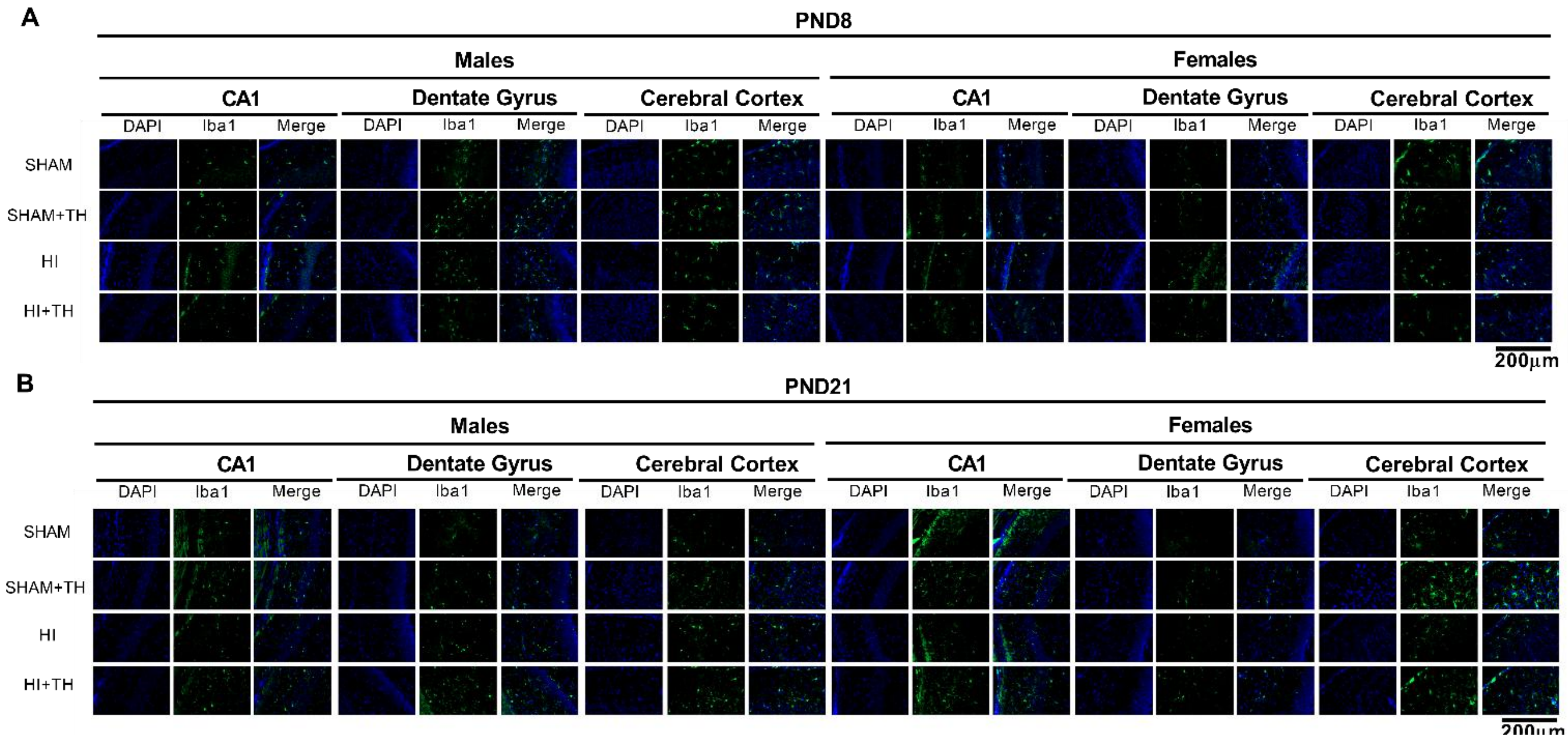

Fig. S2. Microglial reactivity was measured as Iba1 MFI at PND8 or PND21 in male and female pups submitted to hypoxia-ischemia. Representative images of the left (contralateral) brain hemisphere regions analyzed in CA1, dentate gyrus and cerebral cortex are shown at PND8 (A) and PND21 (B). Microglia from all groups showed ramified morphology. For each experimental group of each age, the representative images of the different regions were acquired from the same animal. HI: hypoxia-ischemia, MFI: mean fluorescence intensity; TH: therapeutic hypothermia.

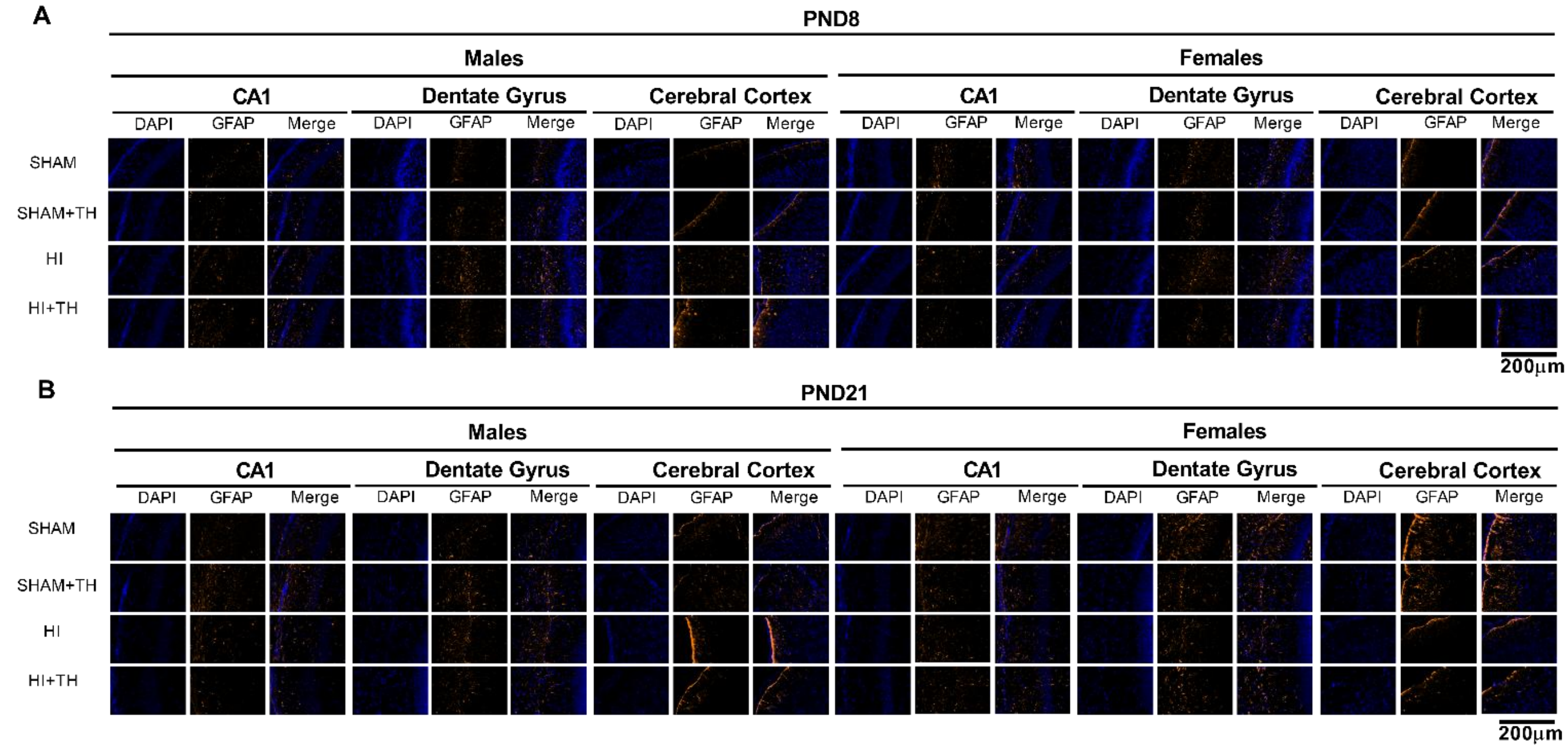

Fig. S3. Astrocytic reactivity was measured as GFAP MFI at PND8 or PND21 in male and female pups submitted to hypoxia-ischemia. Representative images of the left (contralateral) brain hemisphere regions analyzed in CA1, dentate gyrus and cerebral cortex are shown at PND8 (A) and PND21 (B). Astrocytes from all groups did not show a reactive morphology. For each experimental group of each age, the representative images of the different regions were acquired from the same animal. HI: hypoxia-ischemia, MFI: mean fluorescence intensity; TH: therapeutic hypothermia.

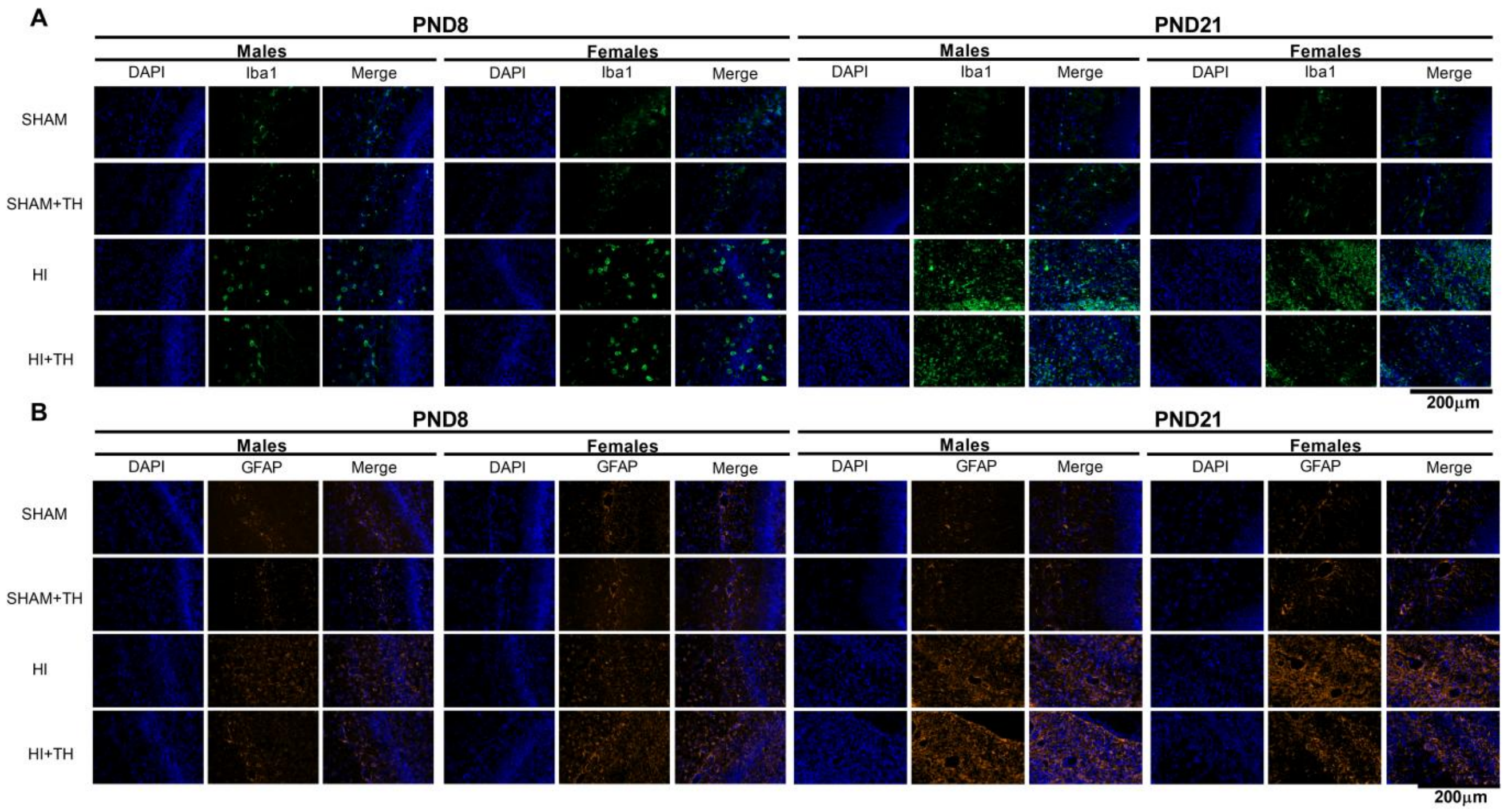

Fig. S4. Microglial and astrocytic reactivity were measured by Iba1 and GFAP MFI, respectively, at PND8 or PND21 in male and female pups submitted to hypoxia-ischemia. Representative images of the right (ipsilateral) dentate gyrus are shown for Iba1 (A) and GFAP (B) labeling. Microglia and astrocytes from the HI and HI+TH groups showed

a reactive morphology. For each experimental group of each age, the representative images of the different regions were acquired from the same animal. HI: hypoxia-ischemia, MFI: mean fluorescence intensity; TH: therapeutic hypothermia.
